# Testing the Empathizing–Systemizing theory of sex differences and the Extreme Male Brain theory of autism in half a million people

**DOI:** 10.1073/pnas.1811032115

**Published:** 2018-11-12

**Authors:** David M. Greenberg, Varun Warrier, Carrie Allison, Simon Baron-Cohen

**Affiliations:** ^a^Autism Research Centre, Department of Psychiatry, University of Cambridge, Cambridge CB2 8AH, United Kingdom

**Keywords:** autism, sex differences, empathy, systemizing, big data

## Abstract

The Empathizing–Systemizing (E-S) theory of typical sex differences suggests that individuals may be classified based on empathy and systemizing. An extension of the E-S theory, the Extreme Male Brain (EMB) theory suggests that autistic people on average have a shift towards a more masculinized brain along the E-S dimensions. Both theories have been investigated in small sample sizes, limiting their generalizability. Here we leverage two large datasets (discovery *n* = 671,606, including 36,648 autistic individuals primarily; and validation *n* = 14,354, including 226 autistic individuals) to investigate 10 predictions of the E-S and the EMB theories. In the discovery dataset, typical females on average showed higher scores on short forms of the Empathy Quotient (EQ) and Sensory Perception Quotient (SPQ), and typical males on average showed higher scores on short forms of the Autism Spectrum Quotient (AQ) and Systemizing Quotient (SQ). Typical sex differences in these measures were attenuated in autistic individuals. Analysis of “brain types” revealed that typical females on average were more likely to be Type E (EQ > SQ) or Extreme Type E and that typical males on average were more likely to be Type S (SQ > EQ) or Extreme Type S. In both datasets, autistic individuals, regardless of their reported sex, on average were “masculinized.” Finally, we demonstrate that D-scores (difference between EQ and SQ) account for 19 times more of the variance in autistic traits (43%) than do other demographic variables including sex. Our results provide robust evidence in support of both the E-S and EMB theories.

## Significance

In the largest study to date of autistic traits, we test 10 predictions from the Empathizing–Systemizing (E-S) theory of sex differences and the Extreme Male Brain (EMB) theory of autism. We confirmed that typical females on average are more empathic, typical males on average are more systems-oriented, and autistic people on average show a “masculinized” profile. The strengths of the study are the inclusion of a replication sample and the use of big data. These two theories can be considered to have strong support. We demonstrate that D-scores (difference between E and S) account for 19 times the variance in autistic traits than do other demographic variables, including sex, underscoring the importance of brain types in autism.

The Empathizing–Systemizing (E-S) theory (1, 2) of sex differences suggests that individuals can be classified on the basis of two dimensions: empathy, defined as the ability to recognize another person’s mental state (“cognitive empathy”) and the drive to respond to it with an appropriate emotion (“affective empathy”) (3), and systemizing, defined as the drive to analyze or build a rule-based system (4). Both of these dimensions are normally distributed in the general population, with well-established biological factors [e.g., prenatal testosterone (5, 6) and common genetic variants (7, 8)] contributing to a proportion of the variance.

The E-S theory makes six predictions to explain typical sex differences in the general population: (*i*) that females on average will score higher on empathy (E) than will males, which has been confirmed (3); (*ii*) that males on average will score higher on systemizing (S) than will females, which has again been confirmed (4, 9); (*iii*) that E and S have a small inverse correlation (4); (*iv*) that, if the data are converted into five “brain types” based on the difference or D-score (S-E) between E and S, such that the brain types are Type B (balanced, where E = S), Type E (where E > S), Type S (where S > E), Extreme Type E (E >> S), and Extreme Type S (S >> E) (4), more females than males will have a brain of Type E; and (*v*) more males than females will have a brain of Type S (these predictions have been confirmed in two modest size samples of fewer than 5,000 people) (4, 10). Additionally, the theory also predicts that, based on differential evolutionary selection pressures on males and females, Type E will have the highest number of females and Type S will have the highest number of males. This prediction has also been confirmed (4, 10). This suggests that evolutionary selection pressures have favored brains that specialize more in one domain than another, in a sex-associated manner, probably because empathy and systemizing are highly adaptive in different environments (social versus technical).

An extension of the E-S theory is the Extreme Male Brain (EMB) theory (11). This proposes that, with regard to empathy and systemizing, autistic individuals are on average shifted toward a more “masculine” brain type (difficulties in empathy and at least average aptitude in systemizing) (11). This may explain why between two to three times more males than females are diagnosed as autistic (12, 13). The EMB makes four further predictions: (*vii*) that more autistic than typical people will have an Extreme Type S brain; (*viii*) that autistic traits are better predicted by D-score than by sex; (*ix*) that males on average will have a higher number of autistic traits than will females; and (*x*) that those working in science, technology, engineering, and math (STEM) will have a higher number of autistic traits than those working in non-STEM occupations.

The two theories and predictions have mostly been tested in relatively small datasets, limiting their generalizability. One large-scale study of autistic traits was conducted by our group using the AQ in half a million people, confirming both sex differences and the STEM effect (14), but no large-scale study has ever tested both the E-S and EMB theories using all three key measures [the Empathy Quotient (EQ), the Systemizing Quotient-Revised (SQ-R), and the Autism Spectrum Quotient (AQ)]. This limits the evidence base of the two theories and has the problems that are typical of small-*n* studies, including, but not limited to, the “winner’s curse” in effect size estimate, sampling bias, and limited statistical power to identify small effects. To address this, we tested the predictions of these two theories in two large independent datasets, with very different recruitment strategies.

## Results

In the discovery dataset, more than 670,000 individuals (including 36,648 autistic individuals) primarily from the United Kingdom completed short versions of the EQ, AQ, SQ-R (henceforth SQ), and a measure of sensory perception (the Sensory Perception Quotient or SPQ) as a part of a Channel 4 TV documentary titled “Are you autistic?” (15). We included the SPQ in light of the new symptom B criteria in the *Diagnostic and Statistical Manual of Mental Disorders* (“Hyper- or hypo-reactivity to sensory input or unusual interest in sensory aspects of the environment”) (16) to examine the role of sensory sensitivity in relation to the E-S and EMB theories.

**Fig. 1.**
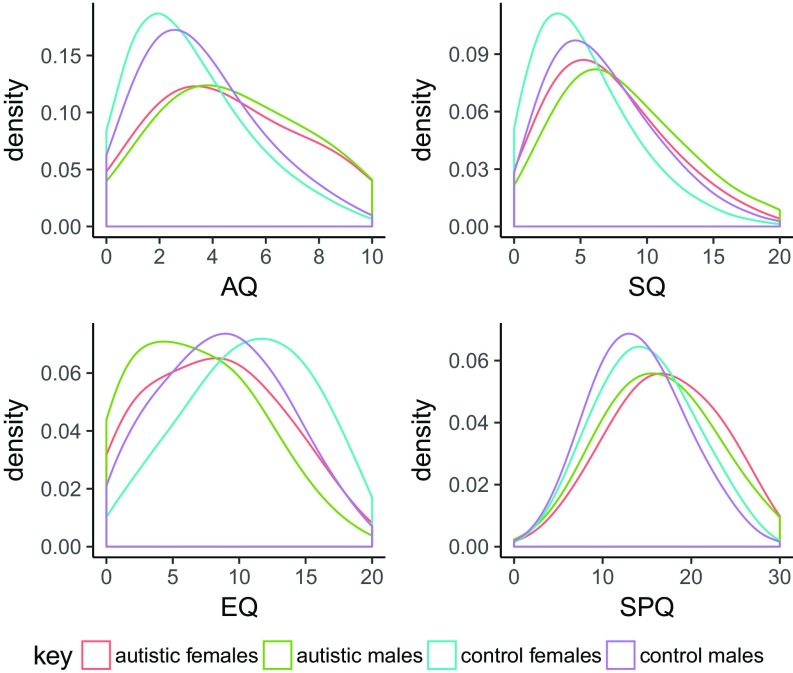
This figure provides the smoothed density plots for all four measures. Each separate graph represents a measure, with scores on the measure provided on the *x* axis. The density is provided on the *y* axis. Each colored line represents a category based on diagnosis and sex.

We first investigated sex differences on the four measures (Table 1). For both autistic individuals (henceforth “cases”) and typical individuals (henceforth “controls”), females on average scored significantly higher on the EQ (Cohen’s D = 0.39, *P* < 2.2 × 10^−16^) (prediction 1) and the SPQ (Cohen’s D = 0.15, *P* < 2.2 × 10^−16^), but males on average scored significantly higher on the SQ (Cohen’s D = 0.31, *P* < 2.2 × 10^−16^) (prediction 2) and the AQ (Cohen’s D = 0.18, *P* < 2.2 × 10^−16^) (prediction 9) (Fig. 1), in line with previous results (3, 4, 14, 17). Effect sizes of sex differences were significantly attenuated for the AQ, the EQ, and the SQ in cases compared with controls (*SI Appendix*, Table S1), in line with previous results (10).

**Table 1.** Means, SDs, and Cohen’s D for sex differences across all measures in cases and controls

Measure	Sex	Controls				Cases				Case–control	
		Mean	SD	Cohen’s D (sex difference)	*P* (sex difference)	Mean	SD	Cohen’s D (sex difference)	*P* (sex difference)	Cohen’s D	*P*
AQ	Males	3.57	2.27	0.18	<2.2 × 10^−16^	4.87	2.66	0.08	7 × 10^−14^	0.52	<2.2 × 10^−16^
	Females	3.16	2.26			4.66	2.74			0.59	<2.2 × 10^−16^
EQ	Males	8.87	4.75	0.39	<2.2 × 10^−16^	6.92	4.71	0.27	<2.2 × 10^−16^	0.41	<2.2 × 10^−16^
	Females	10.79	4.84			8.26	5.03			0.51	<2.2 × 10^−16^
SQ	Males	6.73	4.18	0.31	<2.2 × 10^−16^	8.07	4.64	0.21	<2.2 × 10^−16^	0.30	<2.2 × 10^−16^
	Females	5.45	3.87			7.09	4.37			0.39	<2.2 × 10^−16^
SPQ	Males	13.99	5.51	0.15	<2.2 × 10^−16^	16.33	6.27	0.12	<2.2 × 10^−16^	0.39	<2.2 × 10^−16^
	Females	14.82	5.74			17.10	6.16			0.38	<2.2 × 10^−16^

This table provides the means, the SDs, the effect size estimate of sex differences (Cohen’s D), and the associated *P* value (*t* test) for the four measures (AQ, EQ, SQ, and SPQ) separately for cases and controls. *n* = 241,355 (male controls), 393,600 (female controls), 18,188 (male cases), and 18,460 (female cases). Sex differences were attenuated in cases compared with controls as can be seen from the Cohen’s D.

All four measures were significantly correlated with each other (*P* < 2.2 × 10^−16^). Pearson’s correlation was highest between AQ and EQ (−0.59) and lowest for EQ and SPQ (−0.15). While SQ and SPQ were highly correlated with each other (0.47), there was a small inverse correlation between SQ and EQ (−0.21), suggesting EQ and SQ are largely dissociable dimensions (prediction 3) (Table 2). This correlation is consistent with evidence that social (EQ) and nonsocial (SPQ and SQ) traits related to autism are partly independent (8, 18–22).

**Table 2.** Correlations between the four measures in controls

Measure	AQ	EQ	SQ	SPQ
AQ	1.00	−0.59	0.41	0.34
EQ	−0.59	1.00	−0.21	−0.15
SQ	0.41	−0.21	1.00	0.47
SPQ	0.34	−0.15	0.47	1.00

This table provides the Pearson’s correlation coefficient for pairwise correlations between the four measures in controls (*n* = 634,955). All correlations are highly significant at *P* < 2.2 × 10^−16^. We note that the correlations between the EQ and the SQ and the SPQ are small, though significant. In contrast, the correlation between SPQ and SQ is high.

Correlations between age, education, and the scores on the four measures, although significant, were small, for both cases and controls, and are reported in *SI Appendix*, Tables S2 and S3. For completeness, we investigated if there were geographic differences in the four measures in controls, separately for males and females. Because we had no a priori reasons to investigate geographical differences, and no explanatory framework with which to interpret them, these are simply reported in *SI Appendix*, Table S4. We next investigated if STEM occupation choices showed any association with the four trait measures (*Materials and Methods*). STEM professionals on average scored significantly higher on the AQ (beta = 0.45 ± 0.009, *P* < 2.2 × 10^−16^), the SQ (beta = 1.27 ± 0.016, *P* < 2.2 × 10^−16^), and the SPQ (beta = 0.24 ± 0.022, *P* < 2.2 × 10^−16^), and significantly lower on the EQ (beta = −1.10 ± 0.019, *P* < 2.2 × 10^−16^) (*SI Appendix*, Fig. S1 and Tables S5 and S6) (prediction 10). Autistic individuals were not more likely to work in STEM occupations, compared with controls (Pearson’s χ^2^ test, *P* = 0.59).

To understand how demographics, D-scores, and SPQ are associated with AQ scores, we conducted multiple linear regression analyses with demographics, D-scores, and SPQ as simultaneous predictors of AQ using three regression models in controls (*Materials and Methods* and *SI Appendix*, Table S7). Demographic variables accounted for 2.3% of the variance in model 1: *R*^2^ = 0.023, *P* < 2.2 × 10^−16^. In males, STEM occupations were positively associated with AQ, and age and education were negatively associated with AQ. Handedness and geographic region also showed an effect. In model 2, D-scores accounted for 41.4% of added variance to the model: *R*^2^ = 0.437, *P* < 2.2 × 10^−16^. D-scores were positively associated with AQ, with D-scores as the greatest predictor (prediction 8). Crucially, D-scores accounted for 19 times more of the variance in autistic traits than was accounted for by other demographic variables, including sex, suggesting that brain type is associated with much more of the variance in autistic traits than is sex. In model 3, SPQ accounted for 0.85% of added variance to the model: *R*^2^ = 0.445, *P* < 2.2 × 10^−16^. We further examined if D-scores mediate the effect of sex on AQ (*Materials and Methods*). We found evidence for both direct (beta = 0.308, 95%, CI = 0.30 to 0.32, *P* < 2.2 × 10^−16^) and indirect effects (beta = −0.60, 95%, CI = −0.6 to −0.6, *P* < 2.2 × 10^−16^) of sex on AQ, suggesting that D-scores partially mediate the effect of sex on AQ.

We then examined whether EQ or SQ was driving D-scores as a predictor of AQ. We therefore performed an additional regression analysis with demographics, EQ, SQ, and SPQ entered as predictors of AQ in controls. EQ, SQ, and SPQ together accounted for 46% of added variance to the model: *R*^2^ = 0.459. SQ and SPQ were positively correlated with AQ, and EQ was negatively correlated with AQ. The magnitude of effect was largest for EQ (beta = −0.23, SE = 0.0004, *P* < 2.2 × 10^−16^), followed by SQ (beta = 0.13, SE = 0.0006, *P* < 2.2 × 10^−16^), and then SPQ (beta = 0.05, SE = 0.0004, *P* < 2.2 × 10^−16^).

We investigated differences in sex ratio and scores in cases vs. controls (*Materials and Methods*). We identified a significant difference in the sex ratio between cases and controls, with 1.6 times more males than females reporting having an autism diagnosis (*P* < 2.2 × 10^−16^), in line with epidemiological observations (12, 13). If we classified individuals based on whether they identified as one of the two binary sex options (males or females), versus the nonbinary sex option, 2.5% of autistic individuals in this study identified as nonbinary, compared with only 0.45% from the general population (*P* < 2.2 × 10^−16^). In other words, autistic individuals are 5.5 times more likely to identify as nonbinary compared with the general population, in line with previous findings (23–25). We then investigated if there are differences in scores on the four main measures between cases and controls (*Materials and Methods*). Across all four measures, we found a significant case–control difference, with higher scores on the AQ, SPQ, and the SQ for cases and lower scores on the EQ for cases compared with controls (0.30 < Cohen’s D < 0.59) (Fig. 1 and Table 1). Notably, the effect sizes for case–control differences were larger than the effect sizes for typical sex differences and, with the exception of the SPQ, were higher in female case–control comparisons than to male case–control comparisons.

**Fig. 2.**
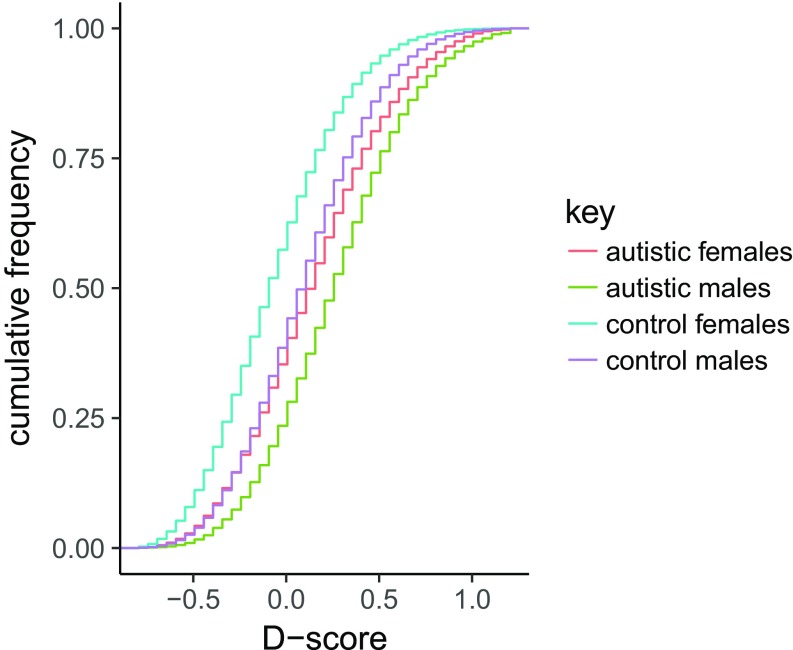
This figure provides the cumulative distribution function based on D-score. The D-score is provided on the *x* axis and the cumulative frequency on the *y* axis. Each colored line represents a category based on sex and diagnosis.

We next investigated differences in brain types (Fig. 2 and Table 3). More females than males were classified as Type E (females 40.00%, males 23.87%) or Extreme Type E (females 2.89%, males 0.75%), and more males were classified as Type S (males 40.23%, females 25.58%) or Extreme Type S (males 4.14%, females 1.69%) (χ^2^ test, *P* < 2.2 × 10^−16^) (predictions 4 and 5). We confirmed that more control females were classified as Type E (observed, 40%; theoretically expected, 32.5%; χ^2^ test, *P* < 2.2 × 10^−16^) while more control males were classified as Type S (observed, 40%; theoretically expected, 32.5%; χ^2^ test, *P* < 2.2 × 10^−16^) (prediction 6). For the autistic male and female groups, the majority of each were classified as Type S (males 50.97%, females 42.29%, χ^2^ test, *P* < 2.2 × 10^−16^ for both sexes), and more autistic people than controls were classified as Extreme Type S (males 11.42%, females 7.55%), with a significant shift toward Type S or Extreme Type S in the autistic group (χ^2^ test, *P* < 2.2 × 10^−16^ for case vs. controls for each sex separately) (prediction 7).

**Table 3.** Frequency distribution of brain types

Brain type	Control males, %	Control females, %	Autistic males, %	Autistic females, %
Extreme Type E	0.75	2.89	0.30	0.93
Type E	23.88	40.01	13.37	22.20
Type B	30.99	29.81	23.92	27.03
Type S	40.24	25.59	50.98	42.29
Extreme Type S	4.15	1.69	11.43	7.55

This table reports the frequency of the control and case populations based on brain types. All numbers are in percentages. *n* = 241,355 (male controls), 393,600 (female controls), 18,188 (male cases), and 18,460 (female cases).

We then investigated if brain types are associated with AQ. There was a significant main effect of brain type on AQ (ANOVA, *P* < 2.2 × 10^−16^). Post hoc Tukey tests showed that all differences between brain types were significant (*SI Appendix*, Table S8). AQ scores were lowest for Extreme Type E (mean = 0.99, SD = 1.00) and increased across the brain types, reaching its height at Extreme Type S (mean = 7.29, SD = 1.95). Second, we conducted an ANOVA with SPQ as the dependent variable (DV) and brain type as the independent variable (IV). There was a significant main effect of brain type on SPQ (*P* < 2.2 × 10^−16^). Post hoc Tukey tests showed that all SPQ differences between brain types were significant. SPQ scores showed the same pattern as for AQ, with SPQ scores lowest for Extreme Type E (mean = 10.36, SD = 5.34) and increasing across the brain types, reaching its highest at Extreme Type S (mean = 20.58, SD = 5.46) (*SI Appendix*, Table S8).

Finally, we conducted a further mediation analysis to test if D-scores mediate the difference in AQ between cases and controls (*Materials and Methods*). We found D-scores do indeed partially mediate the differences in AQ scores between cases and controls and identified both a significant indirect effect (beta = 0.795, 95% CI = 0.78 to 0.80, *P* < 2.2 × 10^−16^) and a smaller, yet significant, direct effect (beta = 0.515, 95% CI = 0.49 to 0.54, *P* < 2.2 × 10^−16^) of case–control status on AQ.

We next investigated if the present findings could be observed in an independent replication cohort that differed in methods and versions of the tests (*Materials and Methods*). Whereas the discovery cohort used the short versions of the four instruments, the replication cohort used full versions of just two of these (EQ and SQ). The data enabled us to test and confirm the first 7 of the 10 predictions. The findings replicated that control females on average scored higher than control males on EQ (Cohen’s D = 0.53, *P* < 2.2 × 10^−16^) (*SI Appendix*, Table S9); control males on average scored higher than control females on SQ (Cohen’s D = 0.19, *P* < 2.2 × 10^−16^) (*SI Appendix*, Table S9); there was a small but nonsignificant inverse correlation between EQ and SQ (r = −0.015, *P* = 0.07), which further supports evidence from genetics and factor analyses that social and nonsocial traits related to autism are largely independent (8, 18, 19). Based on D-score, more females than males were classified as Type E (*SI Appendix*, Table S10) while more males than females were classified as Type S. Further, similar to the discovery dataset, more females were classified as Type E (42.3%, χ^2^ test, *P* < 2.2 × 10^−16^) than were classified as any other type, and more males were classified as Type S (41.07%, χ^2^ test, *P* < 2.2 × 10^−16^) than were classified as any other type. The majority of autistic males (56.83%) and females (60.91%) were classified as Type S (*SI Appendix*, Table S10). Finally, we replicated that more cases have an Extreme Type S brain (*SI Appendix*, Table S10) and that 5.17% of cases identified as a nonbinary sex compared with 0.92% of controls (χ^2^ test, *P* < 0.05). Although the percentage of cases identified as nonbinary is higher in this cohort than in the discovery cohort, the ratios are the same: cases are 5.5 times more likely than controls to identify as a nonbinary sex.

## Discussion

These findings, from the largest dataset to date, confirm all 10 predictions from the E-S and EMB theories, and, where we had the opportunity to test 7 of these in an independent dataset, all of these replicated, testifying to the robustness of these results. The observed average sex differences likely reflect an interaction of biological and cultural factors. Both empathy and systemizing scores are in part explained by exposure levels to fetal testosterone (5, 6) and genetic common variance (7, 8, 26), but this in no way denies the importance of social experience. The brain basis of brain types still needs to be understood, and some studies have begun to map these (27, 28). The present big data also suggest that both autistic males and females show a masculinized shift in terms of being more likely to have a brain of Type S or Extreme Type S. This has relevance for understanding the etiology of autism, implicating a biological mechanism involved in neural sexual dimorphism (11). The EMB theory is in line with brain imaging studies which find that autistic females are masculinized in both brain structure (29, 30) and function (31–33). The EMB theory has also led to studies of sex-linked prenatal etiological factors, such as confirming elevated prenatal sex steroids (34), elevated circulating sex steroids in autistic females (35), and elevated rates of steroidopathy in autistic females, including elevated rates of polycystic ovary syndrome (24, 36).

It is important to address three common misunderstandings about these theories. First, some people are concerned that the EMB theory stereotypes autistic people as having an extreme of all male characteristics (such as aggression). This misunderstanding is likely based on only reading the name of the theory, but not its actual claims. The EMB simply predicts that autistic people on average will show a masculinized pattern of scores on empathy (below average) and systemizing (average or above average), which the current data strongly confirm.

Second, the EMB theory has also been misunderstood as suggesting that autistic individuals lack empathy. However, the lower scores on the EQ likely reflect difficulties primarily with cognitive empathy (or theory of mind), rather than all components of empathy. Experimental studies suggest that affective empathy is intact in autism (37, 38). Individuals with psychopathic/antisocial personality disorder show the opposite dissociation (intact cognitive empathy, and impaired affective empathy), leading to the conclusion that autism and psychopathic/antisocial personality disorder are in some ways mirror opposites of each other (39). Difficulties with cognitive empathy tend to lead autistic people to avoid or be confused by social situations, rather than to act with cruelty (40). Again, the EMB theory deals with averages, and we stress that there is considerable variance in empathic ability in the autistic population.

Finally, the E-S theory has been misunderstood as an example of “neurosexism” by those who wish to dispute that any sex differences in the mind exist (41, 42). However, this is erroneous because the E-S theory does not allow one to make predictions about an individual’s psychological profile based on their biological sex, and to do so would be stereotyping, which is pernicious. The scientific evidence from sex differences research, including the present study, only allows inferences to be drawn about males and females as groups, showing differences on average. This is because an individual may be typical or atypical for their sex. Furthermore, other factors often mediate such sex differences. For example, D-scores mediate sex differences in STEM (43). A careful reading of the E-S theory therefore leads to the conclusion, for example, that it would be wrong to prejudge an applicant for a job in STEM based on their sex, both morally and scientifically.

Limitations of the present study include its reliance on self-report measurements, the risks of convergence across measures, and that we could only include autistic individuals who had the capacity to complete an online survey. It would be worthwhile to replicate these findings based on observer ratings of autistic individuals who are minimally verbal or with intellectual disability, who may be unable to complete a self-report. These limitations are offset by the considerable strengths of the present study: big data, an independent replication cohort, and the opportunity to test two theories comprehensively using multiple measures in the same cohorts. We conclude that the present study provides strong support for both the E-S and EMB theories.

## Materials and Methods

### Discovery Cohort and Analyses: Participants and Procedures.

In Spring 2017, Channel 4 TV developed a website for a documentary later entitled “Are you autistic?” (15). As part of this website, users were able to take several scientific measures and find out how their scores compared with the general population. Participants were asked to provide demographic information and asked to click a checkbox indicating that they would allow their results to be used for scientific research. Only the results of those who checked the box were recorded for the dataset. The website was mobile friendly, and advertisements for the website were placed on the Channel 4 TV website (https://www.channel4.com/). A total of 695,166 participants completed the four measures (see below) and provided demographic information.

Our initial analyses were restricted to participants who indicated they were males or females (672,279). Thus, we removed 22,887 participants who indicated “other” or “prefer not to say” when asked to indicate their sex. Finally, we applied an age cutoff from 16 to 89 y old, to be consistent with other research in the field (14) and removed participants who did not provide their age, leaving 671,606 participants for analysis. Of those who indicated their sex, 259,544 (39%) were male and 412,062 (61%) were female. Their mean age was 29.19 y (SD = 12.20). Of those who indicated, 517,217 (77%) were from the United Kingdom and 154,389 (23%) were from outside of the United Kingdom. The Psychology Research Ethics Committee of the University of Cambridge confirmed that formal ethical review was not needed for use of this dataset since it was secondary use of deidentified and anonymized data.

### Measures.

Participants completed four psychological measures: the Autism Spectrum Quotient-10 (AQ-10) (44) and three newly developed 10-item short forms of the Empathy Quotient (EQ) (3), Systemizing Quotient-Revised (SQ-R) (4), and the Sensory Perception Quotient (SPQ) (45). Development of these short forms is described in *SI Appendix*, and all four measures with their scoring instructions are included in *SI Appendix*. Autistic individuals were identified if they had indicated they had an autism diagnosis either in a question asking about the presence of any clinical diagnosis, or in a separate question asking explicitly if they had an autism diagnosis. In total, there were 36,648 autistic individuals (cases) (18,188 males, 18,460 females). This equates to 5.45% of the sample, which is higher than the population prevalence of autism (1%) (13, 46), possibly due to the nature of the TV program to which this study was linked. Therefore, we restricted several analyses to individuals who did not have a diagnosis of autism (controls) to ensure that the analysis is more representative of the typical population.

In terms of demographics, participants were asked for their sex (“male,” “female,” and “other”), age, occupation [using a list of occupation categories used previously (14)], level of education [“did not complete high school (or A-levels)”], “high school (or A-levels) diploma,” “undergraduate degree,” and “postgraduate degree,” handedness (“right-handed,” “left-handed,” and “ambidextrous”), and geographic location [“Wales,” “Scotland,” “Northern Ireland,” “London (England),” “North East (England),” “North West (England),” “Yorkshire and Humber (England),” “West Midlands (England),” “East Midlands (England),” “South East (England),” “South West (England),” “Other (outside of the United Kingdom),” and “Other (in the United Kingdom)”]. A “prefer not to say” option was provided for all items.

Participants were also asked about any clinical diagnoses they had received. Specifically, participants were presented with nine clinical categories. They were asked to list all of the conditions they had been formally diagnosed with. The options included: “Attention Deficit/Hyperactivity Disorder,” “Autism Spectrum Disorder,” “Bipolar Disorder,” “Depression,” “Learning disability,” “Obsessive-Compulsive Disorder,” and “Schizophrenia.” There was also an option for “I prefer not to say” and “I have not been diagnosed with any of these conditions.” A separate questionnaire item asked those participants who indicated that they had been diagnosed with an “Autism Spectrum Condition” to indicate the exact diagnosis they received, based on the following options: “Autism (classical autism),” “Asperger Syndrome (AS),” and “Other”.

### Calculating Brain Types.

We followed the procedure previously established for calculating E-S brain types (4). Brain type classifications are based on an individual’s D-score, which is the standardized difference of their empathizing and systemizing scores. To calculate the D-score for each participant, first the SQ-R-10 and EQ-10 scores were standardized across the whole sample based on means from the typical population without an autism diagnosis: S = [(SQ-R-10 − <SQ-R-10>)/20 and E = (EQ-10 − <EQ-10>)/20]. That is, we first subtracted the typical population mean using only data from individuals who did not have an autism diagnosis (denoted by <…>) from each individual’s scores, and then divided this by the maximum possible score (20 for the SQ-R-10, and 20 for the EQ-10). The D-score is defined as follows: D = S − E. The brain types were assigned according to the percentiles on the D axis. The lowest scoring 2.5% on the D axis were classified as Extreme Type E and the top 2.5% were classified as Extreme Type S. Those scoring between the 35th and 65th percentile were classified as Type B. Participants who scored between the 2.5th and 35th percentiles were Type E, and Type S was defined by scoring between the 65th and 97.5th percentile. Note that, by definition, only 30% of the population can fall in the Type B category, 32.5% in the Type S and Type E categories individually, and 2.5% in the Extreme Type E and the Extreme Type S categories individually.

### Statistical Analysis.

Statistical analyses were conducted in R version 3.2.3. We first investigated differences in the four 10-item measures using sex and age in cases and controls separately using two-sample *t* tests. Additionally, in controls only, we tested if there were differences in the four measures for handedness and geographical location using ANOVAs. We further investigated the correlations among the four measures in controls and investigated the correlation with educational attainment in controls. We investigated if individuals in STEM occupations are enriched for the four traits using logistic regression in controls. STEM occupation was defined using the same classification used by Ruzich and colleagues (14). We included sex, age, educational attainment, and geographic region as covariates, with STEM status as the independent variable (binary dummy code) and the four trait measures as the dependent variables. To understand how D-scores predict scores on the AQ-10, we conducted multiple regression analysis using two models. In the first model, we included sex (male vs. female), age, handedness (right-handed vs. left-handed), education, and occupation (STEM vs. non-STEM) as predictors of AQ in controls. In the second model, we additionally included D-scores and SPQ to the model, again in controls. Mediation analysis was conducted using the R package mediation (47).

We conducted two mediation analyses. In the first, we investigated if the effect of sex on the AQ is mediated by D-scores in controls. We included country/region, education, age, handedness, and STEM status as covariates in the linear regression. The mediator variable was D-scores, the independent variable was sex [male (coded 0) vs. female (coded 1)], and the dependent variable was AQ scores. In the second, we investigated if differences in AQ scores between cases and controls are mediated by D-scores. To investigate this, we accounted for demographic variables (sex, country/region, education, age, handedness, and STEM status) in the linear regression. The mediator variable was the D-score, the independent variable was case–control status, and the dependent variable was AQ scores.

For case–control analysis, we separated the data into autistic and control groups based on the diagnostic items. A total of 36,648 (18,188 males, 18,460 females) participants indicated that they had a formal diagnosis of autism and were allocated to the autistic group. A total of 634,958 (241,356 males, 393,602 females) indicated that they did not have an autism diagnosis and were allocated to the control group. We conducted χ^2^ tests to investigate if there were differences in sex ratios between cases and controls. Our first χ^2^ test was restricted to males and females. In addition, given that a small fraction of the participants did not identify as either males or females, we conducted a second χ^2^ test using a binary (males or females) and nonbinary (other) classification of sex. The term “Other” here suggests that the participant does not identify as a male or female for social or biological reasons.

### Independent Replication.

To test if the findings replicate, we investigated the first 7 out of the 10 predictions in a second, independent cohort of 14,354 participants (226 autistic individuals, and 14,119 controls). The replication dataset was collected from www.musicaluniverse.org where users completed measures on musical behavior, personality, and cognition, in exchange for feedback about their scores. Participants were directed to the platform from popular media outlets, including CNN and BBC. These participants completed slightly different versions of two of the instruments: the EQ-40 (3) and the 25-item SQ-short (48). The same procedure for calculating brain types and performing statistical analysis used for the initial cohort was also used here.

Participants ranged in age from 18 to 88 y old (mean = 32.38, SD = 12.71). In all, 6,319 (45%) were male and 7,705 (55%) were female. In all, 2,195 (33%) were from the United States, 1,209 (18%) were from the United Kingdom, 474 (7%) were from Germany, and 414 (6%) were from Canada. Therefore, this cohort differed from the first in that it used different recruitment strategies that did not mention autism, they were administered different empathy and systemizing measures, and the participants were more geographically diverse (the majority outside of the United Kingdom). As with the discovery dataset, the Psychology Research Ethics Committee of the University of Cambridge confirmed that formal ethical review was not needed for use of the replication dataset since, again, it was secondary use of deidentified and anonymized data. Given that the maximum scores on the EQ-40 and SQ-25 were different, analyses were conducted using standardized scores for both measures to make them comparable. Analysis scripts, in the form of a knitted document, are available here: https://osf.io/zb6y2/.

### Data and Materials Availability.

Because participants were not asked to consent for their data, even anonymized, to be made publicly available, it is only available on request from those who wish to collaborate with us, via a Visitor Agreement with the University of Cambridge, if appropriate, and under the existing ethical approval. Scripts used to analyze the data are available here: https://osf.io/zb6y2/.

## Supplementary Material

Supplementary File
