# Supplementary material for: Testing the Empathizing–Systemizing theory of sex differences and the Extreme Male Brain theory of autism in half a million people
Source: Proc Natl Acad Sci U S A. 2018 Nov 12;115(48):12152–7. doi: 10.1073/pnas.1811032115 (PMC6275492; doi:10.1073/pnas.1811032115)
Supplement: Supplementary File [file pnas.1811032115.sapp.pdf]

Supplemental Information Appendix

Development of the EQ-10, SQ-R-10, and SPQ-10

Development of the short version of the three measures was carried out independently of autism. Specifically, for all three measures, short versions were developed using scores from individuals without an autism diagnosis.

EQ-10. Prior psychometric analysis has shown that empathy can be measured along a single dimension with the 60-item EQ (1). Wakabayashi et al. (2) developed a brief 22-item version of the EQ. There are four answer options for each item of the EQ: 'strongly agree', 'slightly agree', 'slightly disagree', and 'strongly disagree'. For positively poled items, participants are given 2 points for a 'strongly agree' response, one point for a 'slightly agree' response, and zero points for 'slightly disagree' and 'strongly disagree' responses. This point system is flipped for negatively poled items. To develop the 10-item version of the EQ, we selected the best derived items by calculating the discrimination index (DI) across the 22 items in a sample of 1,494 (747 males and 747 females) who completed the EQ at www.cambridgepsychology.com. These individuals did not have a diagnosis of autism. Specifically, to calculate the DI, we first calculated the frequency distribution of the total EQ scores in the sample. We split the sample into two groups based on the sample's frequency description (high EQ scores [scores of 48 and above] and low EQ scores [scores of 47 and below]). Then for each item, we subtracted the proportion of participants who scored a 1 or 2 in the high EQ group from the proportion of the participants who scored a 1 or 2 in the low EQ group. Good items on a measure are indicated by a discrimination index of 0.3 to 0.7. Based on the DI, the top 10-items naturally consisted of 5 positively poled items and 5 negatively

poled items. The DI for these items are presented in **Table S11** and the correlations between the brief and long measures of the EQ are presented in **Table S12**.

SQ-R-10. Prior psychometric research has shown that the 75-item SQ-R captures a single dimension of systemizing and developed a 44-item gender-neutral version of the SQ-R (3). There are four answer options for each item of the SQ-R: 'strongly agree', 'slightly agree', 'slightly disagree', and 'strongly disagree'. For positively poled items, participants are given 2 points for a 'strongly agree' response, one point for a 'slightly agree' response, and zero points for 'slightly disagree' and 'strongly disagree' responses. This point system is flipped for negatively poled items. To develop a brief 10-item version of the SQ-R we calculated the discrimination index (DI) across the 44 items in a sample of 1,392 typical adults (696 males and 696 females) who completed the SQ-R at www.cambridgepsychology.com. Specifically, to calculate the DI, we first calculated the frequency distribution of the total SQ-R scores in the sample. We split the sample into two groups based on the sample's frequency description (high SQ-R scores [scores of 58 and above] and low SQ-R scores [scores of 57 and below]). Then for each item, we subtracted the proportion of participants who scored a 1 or 2 in the high SQ-R group from the proportion of the participants who scored a 1 or 2 in the low SQ-R group. We then selected the top SQ-R items based on the DI, which consisted of 7 positively poled and 3 negatively poled items. The DI for these items are presented in **Table S13** and the correlations between the brief and long measures of the SQ are presented in **Table S14**.

SPQ-10. The 92-item SPQ measures hyper- and hypo-sensitivity across five subscales: vision, hearing, touch, smell, and taste. Principal component analyses showed that the short 35-

item SPQ indicated that a single dimension consistently assessess sensory hypersensitivity across the five subscales (4). There are four answer options for each item of the SPQ: ‘strongly agree’, ‘agree’, ‘disagree’, and ‘strongly disagree’. For positively poled items, participants are given 3 points for a ‘strongly agree’ response, 2 points for an ‘agree’ response, 1 point for a ‘disagree response’ and zero points for ‘strongly disagree’ response. This point system is flipped for negatively poled items. To develop a brief 10-item version of the SPQ we calculated the discrimination index (DI) across the 35 items in a sample of 428 typical adults (214 males and 214 females) who completed the SPQ at www.cambridgepsychology.com. Specifically, to calculate the DI, we first calculated the frequency distribution of the total 35-item SPQ scores in the sample. We split the sample into two groups based on the sample’s frequency description (high SPQ scores [scores of 44 and above] and low SPQ scores [scores of 43 and below]). Then for each item, we subtracted the proportion of participants who scored a 2 or 3 in the high SPQ group from the proportion of the participants who scored a 2 or 3 in the low SPQ group. We selected the two items with the highest DI for each of the five subscales. For the vision subscale, we replaced item 33 (DI = .37; “I notice the flickering of a desktop computer even when it is working properly”) with item 74 (DI = .33; If I look at a pile of blue sweaters in a shop that are meant to be identical, I would be able to see differences between them) since the former has been outdated with technological advances and retina desktop screens. The DI for these items are presented in Table S5 and the correlations between the brief and long measures of the SPQ are presented in Table S6. For consistency with the scoring options in the EQ-10 and SQ-R-10, for the SPQ-10 we changed the ‘agree’ option to ‘slightly agree’ and the ‘disagree’ option to ‘slightly disagree’. We also constructed the 10-item SPQ so that high scores indicated higher sensory sensitivity. DI is provided in **Table S15**, and correlation between the long and short version is provided in **Table S16. Tables**

S17 – S19 provides the EQ-10, the SQ-10, and the SPQ-10. The AQ-10 is reported in **Table S20** (5)

References

1. Allison C, Baron-Cohen S, Wheelwright SJ, Stone MH, Muncer SJ (2011) Psychometric analysis of the Empathy Quotient (EQ). *Pers Individ Dif* 51(7):829–835.

2. Wakabayashi A, et al. (2006) Development of short forms of the Empathy Quotient (EQ-Short) and the Systemizing Quotient (SQ-Short). *Pers Individ Dif* 41(5):929–940.

3. Allison C, Baron-Cohen S, Stone MH, Muncer SJ (2015) Rasch modeling and confirmatory factor analysis of the systemizing quotient-revised (SQ-R) scale. *Span J Psychol* 18:E16.

4. Tavassoli T, Hoekstra RA, Baron-Cohen S (2014) The Sensory Perception Quotient (SPQ): development and validation of a new sensory questionnaire for adults with and without autism. *Mol Autism* 5(1):29.

5. Allison C, Auyeung B, Baron-Cohen S, Bolton PF, Brayne C (2012) Toward brief "Red Flags" for autism screening: The Short Autism Spectrum Quotient and the Short Quantitative Checklist for Autism in toddlers in 1,000 cases and 3,000 controls [corrected]. *J Am Acad Child Adolesc Psychiatry* 51(2):202–212.e7.

Supplementary Figure 1

Fig. S1. Smoothed density plots of scores on the AQ-10, SQ-10, EQ-10, and SPQ-10 by sex and occupational category (STEM vs. non-STEM)

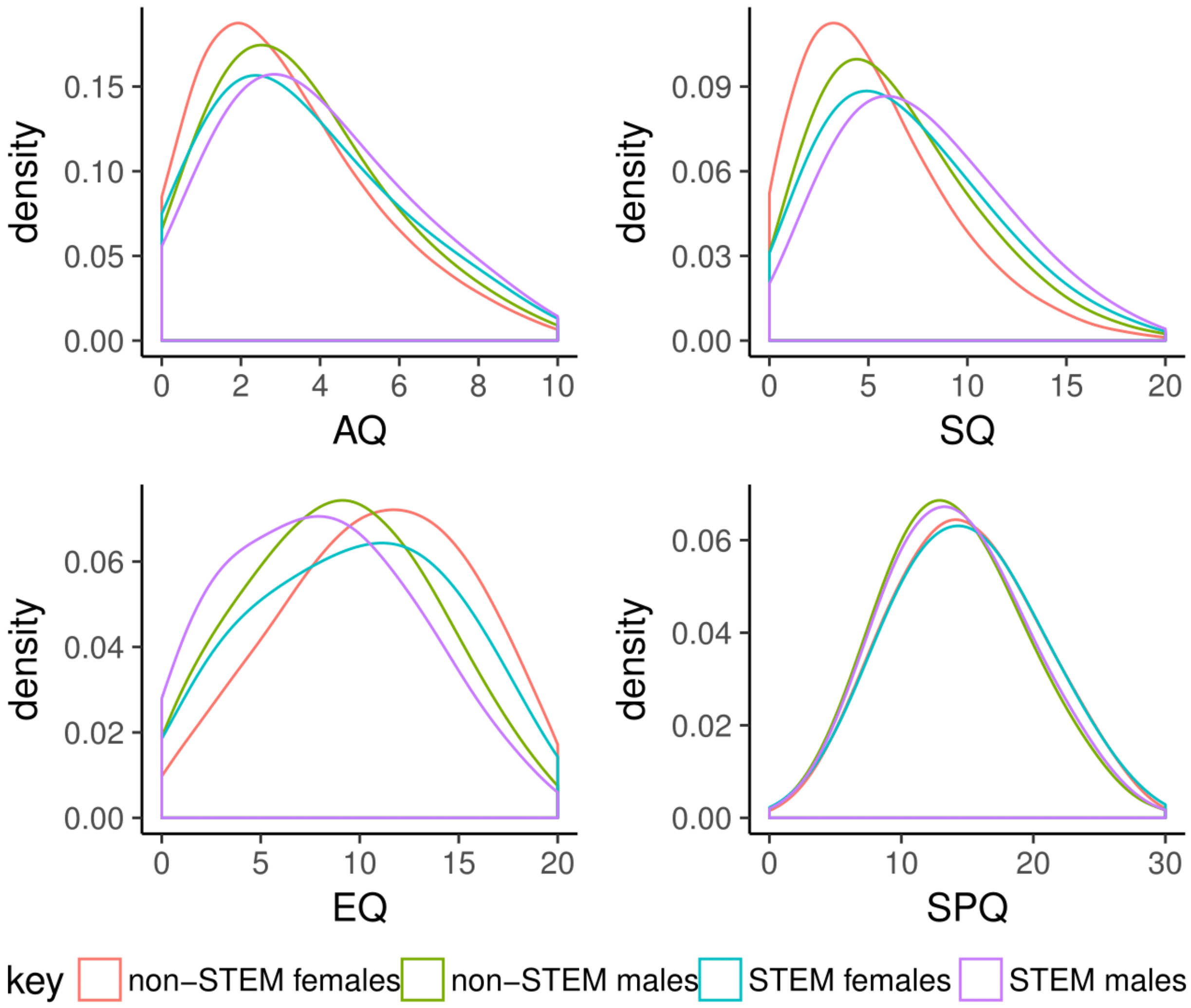

This Figure provides the smoothed density plots for all four measures. Each separate graph represents a measure, with scores on the measure provided on the X-axis. The density is provided on the Y-axis. Each coloured line represents a category based on STEM occupation and sex.

Supplementary Tables S1 – S20

Table S1. Effect size attenuation for sex differences between cases and controls

AQ				
	Estimate	SE	T	P-value
(Intercept)	3.570429	0.004629	771.342	<2e-16
Sex (females vs males)	-0.40116	0.005879	-68.234	<2e-16
Diagnosis (autism vs controls)	1.305203	0.017486	74.644	<2e-16
Sex (females):diagnosis(autism)	0.189616	0.024475	7.747	9.40E-15
EQ				
(Intercept)	8.875432	0.009806	905.12	<2e-16
Sex (females vs males)	1.921288	0.012455	154.26	<2e-16
Diagnosis (autism vs controls)	-1.95494	0.037042	-52.78	<2e-16
Sex (females):diagnosis(autism)	-0.5764	0.051848	-11.12	<2e-16
SQ				
(Intercept)	6.734478	0.008193	822.012	< 2e-16
Sex (females vs males)	-1.28412	0.010406	-123.406	< 2e-16
Diagnosis (autism vs controls)	1.343375	0.030948	43.407	< 2e-16
Sex (females):diagnosis(autism)	0.301009	0.043319	6.949	3.69E-12
SPQ				
(Intercept)	13.99455	0.01159	1207.809	<2e-16
Sex (females vs males)	0.82741	0.01472	56.223	<2e-16
Diagnosis (autism vs controls)	2.336	0.04377	53.37	<2e-16
Sex (females):diagnosis(autism)	-0.05005	0.06126	-0.817	0.414

Table S2. Correlations between four measures and age in cases and controls

	Controls			Cases		
	Correlation coefficient	95%CI	P	Correlation coefficient	95%CI	P
AQ	-0.04	-0.043 to -0.038	< 2.2e-16	0.116	0.106 to 0.126	< 2.2e-16
EQ	0.055	0.052 to 0.057	< 2.2e-16	-0.051	-0.061 to -0.040	< 2.2e-16
SQ	0.038	0.035 to 0.040	< 2.2e-16	0.135	0.125 to 0.145	< 2.2e-16
SPQ	0.1	0.098 to 0.102	< 2.2e-16	0.145	0.135 to 0.155	< 2.2e-16

Table S3. Correlations between the four measures and education in cases and controls

	Controls			Cases		
	Correlation coefficient	95%CI	P	Correlation coefficient	95%CI	P
AQ	-0.09	-0.092 to -0.087	< 2.2e-16	-0.05	-0.060 to -0.039	< 2.2e-16
EQ	0.09	0.097 to 0.102	< 2.2e-16	0.084	0.073 to 0.094	< 2.2e-16
SQ	0.01	0.011 to 0.015	< 2.2e-16	0.022	0.011 to 0.032	2.52E-05
SPQ	-0.07	-0.078 to -0.073	< 2.2e-16	-0.062	-0.072 to -0.052	< 2.2e-16

Table S4. Mean and SD for the four measures in cases and controls in different geographic regions

Sex	Country/region	Measure	Mean	SD	SE of mean
Male	Prefer not to say	AQ	3.555809	2.217204	0.020380817
Male	Prefer not to say	EQ	8.909675	4.660671	0.042841467
Male	Prefer not to say	SQ	7.034643	4.249624	0.039063069
Male	Prefer not to say	SPQ	13.899113	5.713688	0.052520931
Male	Wales	AQ	3.678014	2.304777	0.025550341
Male	Wales	EQ	8.701364	4.655015	0.051604659
Male	Wales	SQ	6.80816	4.197841	0.046536514
Male	Wales	SPQ	14.382205	5.602023	0.062103022
Male	Scotland	AQ	3.514204	2.288172	0.017865442
Male	Scotland	EQ	8.884967	4.707088	0.036751698
Male	Scotland	SQ	6.532553	4.088394	0.031921103
Male	Scotland	SPQ	13.976591	5.418908	0.042309406
Male	Northern Ireland	AQ	3.422964	2.169421	0.026304223
Male	Northern Ireland	EQ	9.206704	4.649885	0.056379853
Male	Northern Ireland	SQ	6.344458	4.00628	0.048576135
Male	Northern Ireland	SPQ	14.069685	5.551684	0.067314162
Male	London	AQ	3.392052	2.210319	0.014566495
Male	London	EQ	9.368686	4.790364	0.031569571
Male	London	SQ	6.470445	4.068623	0.026813134
Male	London	SPQ	13.723474	5.401337	0.035596022
Male	North East	AQ	3.666165	2.307044	0.023120202
Male	North East	EQ	8.627097	4.732503	0.047427109
Male	North East	SQ	6.559707	4.119325	0.041282102
Male	North East	SPQ	14.409862	5.579481	0.055915159
Male	North West	AQ	3.655105	2.301231	0.017212234
Male	North West	EQ	8.708084	4.762919	0.035624614
Male	North West	SQ	6.63228	4.189441	0.03133524
Male	North West	SPQ	14.344112	5.544213	0.041468363
Male	Yorkshire and Humber (England)'	AQ	3.644531	2.29814	0.02027727
Male	Yorkshire and Humber (England)'	EQ	8.542312	4.734909	0.041777702
Male	Yorkshire and Humber (England)'	SQ	6.561386	4.125266	0.036398616
Male	Yorkshire and Humber (England)'	SPQ	14.142468	5.4316	0.047924847
Male	West Midlands (England)	AQ	3.756762	2.317654	0.022117077
Male	West Midlands (England)	EQ	8.634551	4.773413	0.045552062
Male	West Midlands (England)	SQ	6.701211	4.132402	0.039434979
Male	West Midlands (England)	SPQ	14.355887	5.455376	0.052059951
Male	East Midlands (England	AQ	3.780325	2.357122	0.021558838

Male	East Midlands (England	EQ	8.421951	4.775612	0.043678968
Male	East Midlands (England	SQ	6.680107	4.10802	0.037573
Male	East Midlands (England	SPQ	14.333863	5.453611	0.049880121
Male	South East (England)	AQ	3.645673	2.326185	0.013532336
Male	South East (England)	EQ	8.749061	4.811361	0.027989588
Male	South East (England)	SQ	6.578835	4.15807	0.024189141
Male	South East (England)	SPQ	14.117906	5.45744	0.031748086
Male	South West (England)	AQ	3.660664	2.315577	0.017398061
Male	South West (England)	EQ	8.788077	4.781528	0.035925958
Male	South West (England)	SQ	6.707011	4.168076	0.031316793
Male	South West (England)	SPQ	14.214689	5.509498	0.041395558
Male	Other (outside of the United Kingdom)	AQ	3.462366	2.236448	0.009029911
Male	Other (outside of the United Kingdom)	EQ	9.04682	4.740284	0.019139426
Male	Other (outside of the United Kingdom)	SQ	7.056292	4.27511	0.017261236
Male	Other (outside of the United Kingdom)	SPQ	13.593942	5.542732	0.0223794
Male	Other (in the United Kingdom)	AQ	3.628113	2.329412	0.043026727
Male	Other (in the United Kingdom)	EQ	8.814739	4.758338	0.087891593
Male	Other (in the United Kingdom)	SQ	6.757079	4.254652	0.078587977
Male	Other (in the United Kingdom)	SPQ	14.244285	5.673575	0.104797006
Female	Prefer not to say	AQ	3.365892	2.200669	0.018219731
Female	Prefer not to say	EQ	10.484406	4.676465	0.038717287
Female	Prefer not to say	SQ	6.168826	4.020079	0.033282951
Female	Prefer not to say	SPQ	15.317705	5.880741	0.048687702
Female	Wales	AQ	3.256921	2.234406	0.017907365
Female	Wales	EQ	10.636778	4.780182	0.03831017
Female	Wales	SQ	5.469587	3.861338	0.030946212
Female	Wales	SPQ	15.016058	5.757073	0.046139336
Female	Scotland	AQ	3.099083	2.224074	0.01274986
Female	Scotland	EQ	10.895856	4.793614	0.027480158
Female	Scotland	SQ	5.239015	3.774402	0.021637362
Female	Scotland	SPQ	14.559269	5.720422	0.032793232
Female	Northern Ireland	AQ	3.23207	2.171602	0.020651977
Female	Northern Ireland	EQ	10.645745	4.72304	0.044916197
Female	Northern Ireland	SQ	5.310844	3.790276	0.036045595
Female	Northern Ireland	SPQ	14.962648	5.83653	0.055505508
Female	London	AQ	2.966434	2.15489	0.012120479
Female	London	EQ	11.280806	4.846242	0.027258362
Female	London	SQ	5.17881	3.714976	0.020895395
Female	London	SPQ	14.332469	5.664481	0.031860657
Female	North East	AQ	3.228675	2.201043	0.016472086
Female	North East	EQ	10.627779	4.799732	0.03592006
Female	North East	SQ	5.23394	3.781354	0.028298757
Female	North East	SPQ	14.983758	5.707359	0.042712528
Female	North West	AQ	3.197412	2.220658	0.012430764
Female	North West	EQ	10.731739	4.852004	0.027160472
Female	North West	SQ	5.211889	3.78198	0.021170709
Female	North West	SPQ	14.925767	5.696201	0.031886104
Female	Yorkshire and Humber (England)'	AQ	3.225976	2.23534	0.01466404

Female	Yorkshire and Humber (England)'	EQ	10.561088	4.851676	0.031827441
Female	Yorkshire and Humber (England)'	SQ	5.200585	3.735499	0.02450522
Female	Yorkshire and Humber (England)'	SPQ	14.766708	5.629981	0.0369332
Female	West Midlands (England)	AQ	3.185263	2.254703	0.015973938
Female	West Midlands (England)	EQ	10.712694	4.871172	0.034510883
Female	West Midlands (England)	SQ	5.255032	3.785783	0.02682121
Female	West Midlands (England)	SPQ	14.990714	5.721726	0.040536818
Female	East Midlands (England	AQ	3.203944	2.264893	0.015077523
Female	East Midlands (England	EQ	10.669754	4.933196	0.03284057
Female	East Midlands (England	SQ	5.196277	3.765388	0.025066405
Female	East Midlands (England	SPQ	14.899047	5.688854	0.037871029
Female	South East (England)	AQ	3.062481	2.235743	0.009521387
Female	South East (England)	EQ	11.010283	4.921082	0.020957479
Female	South East (England)	SQ	5.070225	3.743741	0.015943522
Female	South East (England)	SPQ	14.765892	5.727821	0.024393149
Female	South West (England)	AQ	3.13666	2.237438	0.012650388
Female	South West (England)	EQ	10.919698	4.888751	0.027640802
Female	South West (England)	SQ	5.222332	3.76813	0.021304855
Female	South West (England)	SPQ	14.826705	5.714171	0.032307698
Female	Other (outside of the United Kingdom)	AQ	3.233928	2.234247	0.007762388
Female	Other (outside of the United Kingdom)	EQ	10.696932	4.825975	0.016766764
Female	Other (outside of the United Kingdom)	SQ	6.178089	4.126008	0.014334888
Female	Other (outside of the United Kingdom)	SPQ	14.851616	5.838423	0.02028429
Female	Other (in the United Kingdom)	AQ	3.311537	2.225395	0.029785994
Female	Other (in the United Kingdom)	EQ	10.463812	4.857625	0.065017323
Female	Other (in the United Kingdom)	SQ	5.539233	3.819782	0.051126223
Female	Other (in the United Kingdom)	SPQ	15.206198	5.802638	0.077665933

Table S5. Mean and SD for the four measures in cases and controls based on STEM groups

sex	STEM	Measure	Mean	SD	SEM
Males	Non-STEM	AQ	3.489074	2.247115	0.005162604
Males	Non-STEM	EQ	9.069335	4.729977	0.010866822
Males	Non-STEM	SQ	6.467117	4.096856	0.009412267
Males	Non-STEM	SPQ	13.952211	5.522411	0.01268739
Males	STEM	AQ	3.867619	2.367948	0.010395341
Males	STEM	EQ	8.167322	4.784522	0.021004151
Males	STEM	SQ	7.711205	4.333073	0.019022278
Males	STEM	SPQ	14.149746	5.489321	0.02409823
Females	Non-STEM	AQ	3.149478	2.216301	0.003627252
Females	Non-STEM	EQ	10.849338	4.831171	0.00790681
Females	Non-STEM	SQ	5.371773	3.839555	0.006283908
Females	Non-STEM	SPQ	14.820487	5.752781	0.009415139
Females	STEM	AQ	3.534249	2.383149	0.016747479
Females	STEM	EQ	9.826609	5.072695	0.035648145
Females	STEM	SQ	6.900044	4.269444	0.030003338
Females	STEM	SPQ	14.850363	5.649177	0.039699353

Table S6. Regression beta and standard error for STEM as a predictor variable for the four measures

	Estimate	Std. Error	P
AQ	0.456418	0.009172	< 2e-16
EQ	-1.10613	0.019208	< 2e-16
SQ	1.271958	0.016184	< 2e-16
SPQ	0.248152	0.022987	< 2e-16

Table S7. Regressions predicting AQ in controls

	Model 1				Model 2				Model 3			
	Estimate	SE	t value	P	Estimate	SE	t value	P	Estimate	SE	t value	P
(intercept)	5.16	0.10	49.36	< 2e-16	3.82	0.08	48.19	< 2e-16	3.30	0.08	41.76	< 2e-16
handedness (Right-handed)	-1.02	0.10	-9.86	< 2e-16	-0.29	0.08	-3.71	0.00	-0.28	0.08	-3.59	0.00
handedness (Left-handed)	-0.96	0.10	-9.23	< 2e-16	-0.28	0.08	-3.55	0.00	-0.27	0.08	-3.39	0.00
handedness (Ambidextrous)	-0.36	0.10	-3.48	0.00	-0.21	0.08	-2.70	0.01	-0.28	0.08	-3.54	0.00
Sex (female)	-0.29	0.01	-48.14	< 2e-16	0.31	0.00	65.79	< 2e-16	0.24	0.00	51.86	< 2e-16
country/region (Wales)	0.07	0.02	3.49	0.00	0.15	0.02	9.57	< 2e-16	0.14	0.02	9.09	< 2e-16
country/region (South East (England))	-0.07	0.02	-4.21	0.00	0.15	0.01	12.26	< 2e-16	0.14	0.01	12.11	< 2e-16
country/region (South West (England))	-0.03	0.02	-1.55	0.12	0.15	0.01	11.40	< 2e-	0.14	0.01	11.07	< 2e-

								16				16
country/region (Other (outside of the United Kingdom))	-0.03	0.02	-1.78	0.07	-0.02	0.01	-2.12	0.03	-0.02	0.01	-1.35	0.18
country/region (Other (in the United Kingdom))	0.04	0.03	1.55	0.12	0.12	0.02	5.62	0.00	0.11	0.02	5.35	0.00
country/region (Scotland)	-0.09	0.02	-5.39	0.00	0.09	0.01	6.67	0.00	0.09	0.01	6.72	0.00
country/region (Northern Ireland)	-0.06	0.02	-2.98	0.00	0.10	0.02	6.19	0.00	0.09	0.02	5.36	0.00
country/region (London (England))	-0.17	0.02	-10.12	< 2e-16	0.08	0.01	6.13	0.00	0.08	0.01	6.20	0.00
country/region (North East (England))	0.02	0.02	1.30	0.19	0.15	0.01	10.61	< 2e-16	0.14	0.01	9.86	< 2e-16
country/region (North West (England))	0.03	0.02	1.72	0.09	0.16	0.01	12.73	< 2e-16	0.15	0.01	11.98	< 2e-16
country/region (Yorkshire and Humber (England))	0.04	0.02	1.96	0.05	0.14	0.01	10.27	< 2e-16	0.14	0.01	10.15	< 2e-16
country/region (West Midlands (England))	0.05	0.02	2.67	0.01	0.18	0.01	12.30	< 2e-16	0.17	0.01	11.75	< 2e-16
country/region (East Midlands (England))	0.06	0.02	3.00	0.00	0.18	0.01	12.84	< 2e-16	0.17	0.01	12.60	< 2e-16

education	-0.22	0.00	-70.51	< 2e-16	-0.11	0.00	-46.83	< 2e-16	-0.10	0.00	-40.82	< 2e-16
age	0.00	0.00	-17.88	< 2e-16	-0.01	0.00	-39.27	< 2e-16	-0.01	0.00	-48.31	< 2e-16
STEM	0.46	0.01	49.76	< 2e-16	-0.06	0.01	-8.30	< 2e-16	-0.04	0.01	-5.28	0.00
D-score					4.31	0.01	684.29	< 2e-16	4.05	0.01	592.71	< 2e-16
SPQ									0.04	0.00	94.31	< 2e-16
	$r^2 = .023$				$r^2 = 0.437$				$r^2 = 0.445$			

This Table reports the results of three-regression models with scores on the AQ-10 as the dependent variable. In the first model, demographics including sex, handedness, age, education, and geographical location have been included. In the second model, additionally, D-scores have been included. In the third model, additionally, scores on the SPQ have been included. None of the variables have been standardized. Estimate is the regression coefficient, SE is the standard error of the regression coefficient, and P is the associated P-value. The variance explained is provided at the bottom (r^2). Several variables (sex, handedness, STEM degree, and country/region) have been coded as categorical variables. For country-region and handedness, this included a 'prefer not to say' option and this too has been included as a categorical variable. Regression coefficients are provided for the category in parenthesis in comparison with another category. For sex, the comparison is males, for handedness the comparison is 'prefer not to say', for STEM degree, it is non-STEM degree, and for country/region, it is 'prefer not to say'. We included 'prefer not to say' as a category as it may be a non-random choice. Continuous variables like education, age, D-score and SPQ, have been coded from lower to higher values. Negative betas suggest a negative correlation between the continuous variable and the AQ and vice versa. Lower D-scores indicate higher scores on the EQ compared to the SQ.

Table S8. AQ and SPQ scores for the four brain types

Brain type	Category	Mean	Standard deviation	Standard error of mean
Extreme Type E	AQ	0.991822	1.002425	0.008723
Type E	AQ	1.935857	1.432427	0.003089
Type B	AQ	3.107094	1.744665	0.00398
Type S	AQ	4.857365	2.135523	0.004801
Extreme Type S	AQ	7.298873	1.953192	0.015125
Brain type	Category	Mean	Standard deviation	Standard error of mean
Extreme Type E	SPQ	10.36332	5.345941	0.04652
Type E	SPQ	12.53279	5.241795	0.011302
Type B	SPQ	14.28662	5.244543	0.011965
Type S	SPQ	16.6333	5.410782	0.012165
Extreme Type S	SPQ	20.58449	5.469007	0.042351

Table S9. Mean EQ and SQ scores in the validation dataset

Category	Variable	Mean	SD	SEM	N	Cohen's D (sex, cases)	
Autism (females)	EQ	28.72414	12.890135	1.3819675	87		
Autism (males)	EQ	26.1295	11.359625	0.96351082	139	0.21	
Autism (females)	SQ	17.09195	4.392468	0.47092196	87		
Autism (males)	SQ	16.08633	4.036935	0.34240832	139	0.23	
Category	Variable	Mean	SD	SEM	N	Cohen's D (sex, controls)	Cohen's D (case-control)
Control (females)	EQ	44.14328	12.543886	0.14219571	7782		1.21
Control (males)_	EQ	37.65015	11.881101	0.14925018	6337	0.53	0.99
Control (females)	SQ	15.30995	4.040923	0.04580733	7782		0.42
Control (males)	SQ	16.08237	3.686735	0.0463127	6337	0.199	0.001

Table S10. Brain type by sex in the validation dataset

Brain type	Control Males	Control Females	Autistic Males	Autistic Females
Extreme Type S	3.85	1.81	20.86	18.39
Type S	41.07	23.83	56.83	60.91
Type B	32.17	28.72	15.1	10.34
Type E	22.43	42.31	7.19	10.34
Extreme Type E	0.45	3.31	0	0

Table S11. Discrimination Index for the EQ-10 items

	**DI (%)**
35. I can easily work out what another person might want to talk about.	.45
12. It is hard for me to see why some things upset people so much.	.42
22. Other people tell me I am good at understanding how they are feeling and what they are thinking.	.42
18. I can't always see why someone should have felt offended by a remark.	.41
34. I can tune into how someone else feels rapidly and intuitively.	.41
14. I am good at predicting how someone will feel.	.39
4. I find it hard to know what to do in a social situation.	.37
9. In a conversation, I tend to focus on my own thoughts rather than on what my listener might be thinking.	.37
31. Other people often say that I am insensitive, though I don't always see why.	.37
28. Friends usually talk to me about their problems as they say that I am very understanding.	.36

Table S12. Correlations between brief and long forms of the EQ

	EQ-40	EQ-22	EQ-10
EQ-40	1	.954**	.935**
EQ-22	.954**	1	.959**
EQ-10	.935**	.959**	1
n = 2,968			

Table S13. Discrimination Index for the SQ-R-10 items

Item	DI (%)
32. When I learn about a new category I like to go into detail to understand the small differences between different members of that category.	43.3
16. When I'm in a plane, I do not think about the aerodynamics.	39.3
27. I am interested in knowing the path a river takes from its source to the sea.	39.2
9. When travelling by train, I often wonder exactly how the rail networks are coordinated.	38.1
30. When I hear the weather forecast, I am not very interested in the meteorological patterns	33.8
33. I enjoy looking through catalogues of products to see the details of each product and how it compares to others	33.7
12. When I look at a mountain, I think about how precisely it was formed	33.5
25. When I look at a piece of furniture, I do not notice the details of how it was constructed.	32.2
8. When I learn a language, I become intrigued by its grammatical rules	32.1
7. When I listen to a piece of music, I always notice the way it's structured.	31.5

Table S14. Correlations between brief and long forms of the SQ-R

	SQ-75	SQ-44	SQ-10
SQ-75	1	.936**	.830**
SQ-44	.936**	1	.867**
SQ-10	.830**	.867**	1
n = 2,774			

Table S15. Discrimination Index for SPQ-10 items

Item	Subscale	Item	DI%
62	smell	I would be able to smell the smallest gas leak from anywhere in the house.	.41
58	taste	I would be able to taste the difference between apparently identical pieces of candy.	.39
88	vision	I can see dust particles in the air in most environments.	.39
2	smell	I would be able to distinguish different people by their smell.	.38
73	hearing	I would be the first to hear if there was a fly in the room.	.35
74	vision	If I look at a pile of blue sweaters in a shop that are meant to be identical, I would be able to see differences between them.	.33
38	touch	I would be able to tell the weight difference between two different coin sizes on the palm of my hand, if my eyes were closed.	.33
35	touch	I would be able to notice a tiny change (for example, 1 degree) in the temperature of the weather.	.33
32	hearing	I can hear electricity humming in the walls.	.30
21	taste	I would be able to taste the difference between two brands of salty potato chips/crisps.	.21

Table S16. Correlations between brief and long forms of the SPQ

	SPQ-92	SPQ-35	SPQ-10
SPQ-92	1	.934**	.828**
SPQ-35	.934**	1	.920**
SPQ-10	.828**	.920**	1
n = 1,711			

Table S17: The Empathy Quotient-10 (EQ-10)

		strongly agree	slightly agree	slightly disagree	strongly disagree
1.	I am good at predicting how someone will feel.	2	1	0	0
2.	Other people tell me I am good at understanding how they are feeling and what they are thinking.	2	1	0	0
3.	It is hard for me to see why some things upset people so much.	0	0	1	2
4.	I can easily work out what another person might want to talk about.	2	1	0	0
5.	I can't always see why someone should have felt offended by a remark.	0	0	1	2
6.	I can tune into how someone else feels rapidly and intuitively.	2	1	0	0
7.	Other people often say that I am insensitive, though I don't always see why.	0	0	1	2
8.	In a conversation, I tend to focus on my own thoughts rather than on what my listener might be thinking.	0	0	1	2
9.	Friends usually talk to me about their problems as they say that I am very understanding.	2	1	0	0
10.	I find it hard to know what to do in a social situation.	0	0	1	2

Table S18: The Systemizing Quotient-Revised-10 (SQ-R-10)

		strongly agree	slightly agree	slightly disagree	strongly disagree
1.	When I learn about a new category I like to go into detail to understand the small differences between different members of that category.	2	1	0	0
2.	When I'm in a plane, I do not think about the aerodynamics.	0	0	1	2
3.	I am interested in knowing the path a river takes from its source to the sea.	2	1	0	0
4.	When travelling by train, I often wonder exactly how the rail networks are coordinated.	2	1	0	0
5.	When I hear the weather forecast, I am not very interested in the meteorological patterns.	0	0	1	2
6.	I enjoy looking through catalogues of products to see the details of each product and how it compares to others.	2	1	0	0
7.	When I look at a mountain, I think about how precisely it was formed.	2	1	0	0
8.	When I look at a piece of furniture, I do not notice the details of how it was constructed.	0	0	1	2
9.	When I learn a language, I become intrigued by its grammatical rules.	2	1	0	0
10.	When I listen to a piece of music, I always notice the way it's structured.	2	1	0	0

Table S19: The Sensory Perception Quotient-10 (SPQ-10)

		strongly agree	slightly agree	slightly disagree	strongly disagree
1.	I would be able to distinguish different people by their smell.	3	2	1	0
2.	I would be able to taste the difference between two brands of salty potato chips/crisps.	3	2	1	0
3.	I can hear electricity humming in the walls.	3	2	1	0
4.	I would be able to notice a tiny change (for example, 1 degree) in the temperature of the weather.	3	2	1	0
5.	I would be able to taste the difference between apparently identical pieces of candy.	3	2	1	0
6.	I would be able to tell the weight difference between two different coin sizes on the palm of my hand, if my eyes were closed.	3	2	1	0
7.	I would be able to smell the smallest gas leak from anywhere in the house.	3	2	1	0
8.	I would be the first to hear if there was a fly in the room.	3	2	1	0
9.	If I look at a pile of blue sweaters in a shop that are meant to be identical, I would be able to see differences between them.	3	2	1	0
10.	I can see dust particles in the air in most environments.	3	2	1	0

Table S20: Autism Spectrum Quotient-10 (AQ-10)

		definitely agree	slightly agree	slightly disagree	definitely disagree
1.	I often notice small sounds when others do not	1	1	0	0
2.	I usually concentrate more on the whole picture, rather than the small details.	0	0	1	1
3.	I find it easy to do more than one thing at once.	0	0	1	1
4.	If there is an interruption, I can switch back to what I was doing very quickly.	0	0	1	1
5.	I find it easy to 'read between the lines' when someone is talking to me.	0	0	1	1
6.	I know how to tell if someone listening to me is getting bored.	0	0	1	1
7.	When I'm reading a story I find it difficult to work out the characters' intentions.	1	1	0	0
8.	I like to collect information about categories of things (e.g. types of car, types of bird, types of train, types of plant etc).	1	1	0	0
9.	I find it easy to work out what someone is thinking or feeling just by looking at their face.	0	0	1	1
10.	I find it difficult to work out people's intentions.	1	1	0	0
